# Performance of *Trichogramma japonicum* under field conditions as a function of the factitious host species used for mass rearing

**DOI:** 10.1371/journal.pone.0256246

**Published:** 2021-08-19

**Authors:** Basana Gowda G., Guru Pirasanna Pandi G., Farman Ullah, Naveenkumar B. Patil, Madhusmita Sahu, Totan Adak, Somnath Pokhare, Manoj Kumar Yadav, Annamalai Mahendiran, Priyanka Mittapelly, Nicolas Desneux, Prakash Chandra Rath

**Affiliations:** 1 Division of Crop Protection, ICAR-National Rice Research Institute, Cuttack, Odisha, India; 2 Department of Entomology, College of Plant Protection, China Agricultural University, Beijing, China; 3 ICAR- National Research Centre on Pomegranate, Solapur, Maharashtra, India; 4 USDA APHIS PPQ, Brighton, MI, United States of America; 5 Université Côte d’Azur, INRAE, CNRS, UMR ISA, Nice, France; King Khalid University, SAUDI ARABIA

## Abstract

Different factitious hosts were used to mass rear *Trichogramma japonicum* Ashmead in different parts of the globe because thorough details were lacking in both the laboratory and the field. The objective of this study was to compare, parasitoid, *T*. *japonicum* reared in different factitious hosts. Three commonly used factitious host eggs, *Corcyra cephalonica* (Stainton), *Ephestia kuehniella* Zeller and *Sitotroga cerealella* Olivier were tested under laboratory conditions and then in the field over a yellow stem borer, *Scirpophaga incertulus* (Walker) of rice. The highest parasitism by *T*. *japonicum* was observed on *E*. *kuehniella* eggs. The parasitoid’s highest emergence (88.99%) was observed on *S*. *cerealella* eggs at 24 h exposure, whereas at 48 h it was on *E*. *kuehniella* eggs (94.66%). *Trichogramma japonicum* females that emerged from *E*. *kuehniella* eggs were significantly long-lived. The days of oviposition by hosts and the host species were significant individually, but not their interaction. Higher proportions of flying *T*. *japonicum* were observed when reared on *E*. *kuehniella* and *C*. *cephalonica* eggs. Field results showed that *T*. *japonicum* mass-reared on *E*. *kuehniella* showed higher parasitism of its natural host, *S*. *incertulus* eggs. Hence, by considering these biological characteristics and field results, *E*. *kuehniella* could be leveraged for the mass rearing of quality parasitoids of *T*. *japonicum* in India, the Asian continent and beyond.

## 1. Introduction

Insect pests continue to influence agricultural production and human health significantly [1]. Chemical control of pests is a common practice to minimize crop losses [2–4]; still, their impacts on the soil, environment, non-target organisms, and the presence of residues in food products are matters of global concern [5–7]. Furthermore, insecticide-induced resistance and hormesis are thought to be significant factors in the failure of chemical-based insect pest control [8–12]. Alternatively, biological control agents have the potential to reduce crop losses and the use of insecticides [13–16]. Of the various bioagents, the family Trichogrammatidae (Order: Hymenoptera) has few successful biological control agents for agricultural and forest pests [17,18], the most important genus under the family being *Trichogramma* [19]. Several *Trichogramma* species are widely used commercially to control lepidopteran pests on economically important crops [20]. Globally, *Trichogramma* spp. is a mainstay of augmentative biological control and is annually applied to 15 million hectares across 40 countries [19]. *Trichogramma* is widely released in India to suppress different caterpillar pests attacking rice, maize, sugarcane, cotton, vegetables, and fruits. The short generation time and mass rearing on factitious hosts allow *Trichogramma* to be produced quickly and affordably compared to other parasitoids [19,21].

Rice (*Oryza sativa* L.) is the staple food for 2.5 billion people worldwide [22]. The rice crop is prone to biotic stress throughout the crop growth period. Among various insect pests that infest rice, yellow stem borer (YSB), *Scirpophaga incertulas* (Walker) (Lepidoptera: Crambidae) is a major pest and has the potential to cause loss up to 87.66% [23]. Apart from insecticides, *T*. *japonicum* Ashmead has been used extensively to manage YSB [19,24]. *Trichogramma japonicum* release can increase grain yields by 25.79%−45.13% over insecticide application [25]. However, despite the considerable release of *T*. *japonicum* in the field, consistent results are often not obtained due to parasitoid quality variability [26]. The field success of *Trichogramma* spp. species depends on selecting suitable host species, their maintenance, etc. Many good quality parasitoids (~300,000 parasitized eggs/ha) are needed to manage YSB in the field [26].

A better understanding of the development and behaviors of parasitoids on various factitious hosts is essential to explore their potential as biological control agents of crop pests [27,28]. Several studies reported sub-optimum parasitoid quality in India due to several factors during their mass rearing [29]. Mass rearing of *T*. *japonicum* in the laboratory on a suitable factitious host is the critical factor determining the quality of the parasitoids. Different factitious hosts are used to mass rear *T*. *japonicum* in different parts of the globe, typically based on the convenience and local availability of hosts and their diets. Therefore, the selection of suitable factitious hosts based on the quality of reared parasitoids has remained vital. Keeping this in mind, the objective of the present study was to investigate the productivity and efficiency of *T*. *japonicum* reared on eggs of three factitious hosts, *Corcyra cephalonica* (Stainton) (Lepidoptera: Pyralidae), *Ephestia kuehniella* Zeller (Lepidoptera: Pyralidae), and *Sitotroga cerealella* Olivier (Lepidoptera: Gelechidae). The performance of parasitoids was assessed using various methods such as parasitism rate, fecundity, offspring sex ratio, longevity, etc. in the laboratory and correlated with the efficiency of parasitizing a target host under field conditions. The present study evaluated the biological parameters of *T*. *japonicum* reared in three different hosts and their field efficiency to control YSB.

## 2. Materials and methods

### 2.1 Study environment

Study was carried out under controlled conditions (Temperature: 25 ± 1°C, relative humidity: 70 ± 10%, and 12 h photophase). Parameters were measured with an indoor thermometer/hygrometer (Model: HTC-1; Zhejiang Junkaishun Industrial & Trade Co Ltd., Zhejiang, China).

### 2.2 Insects

Host insects, *C*. *cephalonica*, *E*. *kuehniella*, *S*. *cerealella* and the parasitoid, *T*. *japonicum* were maintained in insect growth chambers (Daihan Labtech India Pvt. Ltd; LGC 5201 model). The *C*. *cephalonica* eggs and *T*. *japonicum* pupae were collected from ICAR-National Bureau of Agricultural Insect Resources, Bengaluru, India. Initial colony of the other two host species was obtained from the Grain Entomology laboratory, ICAR-NRRI, Cuttack, India. *Corcyra cephalonica* was reared using a maize diet [30]. The larvae of *E*. *kuehniella* were reared as per Lima et al. [31], and *S*. *cerealella* was reared on a susceptible rice variety, TN-1 [32]. To rear *T*. *japonicum*, factitious host eggs were UV-irradiated (30 W UV tube for 45 min at a distance of 45 cm) in a sterilization chamber using UV-C lamp (HNS 15W G13; Osram Puritec, Russia). Host eggs were glued on paper cards (15 × 10 cm). These eggs were exposed to the female wasp in the ratio of 40:1 until adult mortality. The population was maintained in tubes kept in insect growth chambers set at a temperature: 25 ± 1°C, relative humidity: 65 ± 10% RH with 14-h photophase and were used in the different experiments. For all the insect samples used in the study, no permission was required for the insect sampling and collection. Permission was not required because the insects used for the study are common insect pests. The insects used for this study are not endangered species.

### 2.3 Parasitism

Freshly collected eggs of *C*. *cephalonica*, *E*. *kuehniella*, and *S*. *cerealella* (0 to 4 h old) were UV irradiated as described above to arrest their development. Thirty eggs were pasted on a thick yellow paper card (2×2 cm) with gum Arabic (50%) diluted in distilled water. Twenty biological replicates were maintained with 30 eggs per host, and each replicate corresponded to an acrylic tube (6 cm height × 3.5 cm diameter) covered with a sterilized cotton plug. Cards of different host eggs were exposed to a mated adult female of *T*. *japonicum* (less than 24 h of age) individualized in an acrylic tube which had a drop of pure honey on the inner wall as a food source. A similar experiment was conducted for unmated females as well. Two sets of exposure durations of parasitoid to host eggs were maintained. Female *T*. *japonicum* was allowed to parasitize for 24 h, while in another, it was allowed for 48 h. All replicates were placed in an insect growth chamber as described in the previous section. Eggs were observed daily using a microscope (Nikon SMZ-745T, Japan). After the exposure period, the parasitoid was removed and discarded. The rate of parasitism of host eggs was assessed by recording the number of eggs turning black (> 5 days after development), indicating parasitism. Further, percent parasitism was calculated based on the number of blackened eggs among the total number of exposed eggs (30 eggs). The emergence rate was determined by recording the number of emergence holes from the black eggs using a microscope at 40X magnification.

### 2.4 Longevity

Longevity experiments consisted of two treatments based on the presence and absence of honey as food to parasitoids. Once the parasitoids emerged from the tested factitious host eggs, 20 females were randomly selected from each treatment for the experiment, corresponding to twenty biological replicates. Parasitoids were kept individually in a clean glass test tube (7.5 cm height × 1 cm diameter) containing a droplet of honey and covered with parafilm. Similar set up devoid of honey comprised ‘without food’ treatment. The entire setup was kept in a growth chamber as per the conditions described before. Observations were recorded at 24 h intervals from the day of emergence until parasitoids died. Further, the survival rate of parasitoids was calculated, and comparisons were made among different host eggs used.

### 2.5 Parasitism *vs*. days of oviposition by moths

To assess whether egg quality varies with the day of oviposition, eggs of each of the factitious hosts were collected daily from the first day till the 6^th^ day of oviposition for each host separately. Egg cards having 30 eggs were prepared for each day from each host. Ten replicates were maintained. Cards were exposed to parasitism by a freshly emerged parasitoid (<24 h old) described in section 2.3. Eggs selected were of similar age (<12 h post-oviposition). All replicates were placed in a growth chamber. The data on parasitism and parasitoid emergence was recorded.

### 2.6 Flight test

Flight capacity of the adult parasitoids reared on the different host eggs was conducted as per the methodology of Dutton and Bigler [33] with slight modification. In brief, a testing unit (S1 Fig) was comprised of a glass jar (20.5 cm height × 15 cm diameter) lined with a black colored sheet on its inner wall. On the inner side of this sheet, 0.5 cm thick ring of glue (metroark; Wacker Metropark Chemicals Pvt. Ltd., India) was applied, which was 3.5 cm from the bottom of the jar to trap the parasitoids walking upward (walkers). Similarly, the flying parasitoids (flyers) were trapped by covering the top of the jar covered with a Petri dish (15 cm diameter), with glue on their inner surface. A card containing approximately 180 parasitized eggs with a pure drop of honey was placed at the bottom of a glass vial (7.5 cm height × 2.5 cm diameter) in the center of the jar from where wasps emerged. Five replications were maintained for each host. The entire setup was placed in growth chamber conditions, as mentioned earlier. Wasps caught in the glue in the Petri dish designated as ‘flyers’; wasps caught in the lower glue of band (3.5 cm from the bottom) designated as ‘walkers’ and wasps found below lower glue of band designated as ‘non-flyers’. Numbers in each of these locations within the test unit were expressed in percentage.

### 2.7 Field studies

The field performance of *T*. *japonicum* reared on different factitious host eggs was tested by releasing the wasps in the paddy field. Multi-location field studies were conducted at the research farm, ICAR-National Rice Research Institute (NRRI), Cuttack during monsoon (fall harvest) 2019, and at a farmer’s field, Mahanga, during monsoon (fall harvest) 2020 with the rice variety, Pooja. Regarding field site access, NRRI, Cuttack field was our institute experimental farm, hence no permission was required. Whereas, other site was a farmer field, access was obtained with mutual understanding. Four treatments (*T*. *japonicum* reared on three different factitious host eggs and untreated control), and five replications were maintained. All recommended cultural practices were applied until harvest (excluding pesticide applications). ‘Trichocards’ containing *T*. *japonicum* parasitized eggs were placed, corresponding to release densities of 50,000 male and female wasps/ha. Trichocards were prepared using three different factitious host eggs. Each release point was separated by 10 m within the plot. Each plot covered 400 m^2^ and was at least 15 m (edge to edge) away from the neighboring treatment plots [34]. Tricho cards were placed in an inverted, transparent plastic cup fixed to a wooden pole to avoid direct rainfall. When the eggs were blackened (ready-to-emerge), tricho-cards were released, and sentinel YSB egg masses less than 24 h old were placed in the plots. The sentinel egg masses were obtained from the glasshouse cage rearing. For each plot, 10 YSB egg masses with a rice leaf segment was randomly fixed on leaf by a stapler. YSB egg masses were retrieved from the fields after 48 hours and taken back to the laboratory individually on a moistened filter paper in a plastic vial (8.0 cm high × 2.0 cm in diameter) and observed periodically for the emergence of adult parasitoids. From each egg mass, number of hatched YSB larvae, emerged wasps, and dead eggs were recorded. Egg parasitism was calculated by dividing the number of parasitized eggs with total eggs and expressed in percentage.

### 2.8 Data analyses

The student’s t-test was for comparing two treatments and the one-way ANOVA was used (P < 0.05) when there were more than two treatments. Two-way ANOVA was performed to analyze the interaction of the day of oviposition by the host and different species of host. Treatment means were compared based on Tukey’s honest significant difference test (HSD) at 0.05-probability level. Analyses were performed using SAS software (Version 9.4). Data are shown as means ± SD based on data from replicate experiments.

## 3. Results

### 3.1 Parasitism

Parasitisation percentage of mated *T*. *japonicum* varied among the duration parasitisation time (for *C*. *cephalonica*: t = -10.271, df = 19, *P*<0.001; for *E*. *kuehniella* t = -6.343, df = 19, *P*<0.001; for *S*. *cerealella*: t = -11.653 and df = 19) and also the factitious host eggs used (24 h exposure: F_2,57_ = 14.23, *P*<0.0001; 48 h exposure: F_2,57_ = 6.94, df = 38, *P*<0.0001). When suitability of factitious hosts for parasitization was compared, *E*. *kuehniella* recorded an increased 23.74% and 32% parasitization at 24 h exposure and 12.33% and 22.17% at 48 h exposure over *C*. *cephalonica* and *S*. *cerealella*, respectively. Similarly, when exposure duration was increased from 24 h to 48 h, an additional 24.91%, 13.50%, and 23.33% parasitization was observed in *C*. *cephalonica*, *E*. *kuehniella*, and *S*. *cerealella* eggs, respectively (Table 1).

**Table 1:** Parasitism and emergence of *Trichogramma japonicum* on different factitious host eggs, when host eggs were exposed to mated and unmated female parasitoids for 24 and 48 h.

Host	Parasitism (%)				Emergence (%)			
	Mated		Unmated		Mated		Unmated	
	24 h	48 h	24 h	48 h	24 h	48 h	24 h	48 h
***Corcyra cephalonica***	48.67±1.54^Bb^	73.67±1.89^Ba^	40.17±1.54^Bb^	72.83±1.21^Ba^	58.30±3.20^Cb^	86.00±1.18^Ba^	42.29±2.72 ^Cb^	52.84±3.48 ^Ba^
***Ephestia kuehniella***	72.50±1.34^Ab^	86.00±1.65^Aa^	55.67±1.41^Ab^	80.33±1.21^Aa^	65.84±2.35^Bb^	94.66±0.67^Aa^	48.34±1.98 ^Ab^	71.44±2.34 ^Aa^
***Sitotroga cerealella***	40.50±1.42^Cb^	63.83±1.42^Ca^	34.17±0.87^Cb^	45.33±1.12^Ca^	88.99±1.68^Ab^	82.39±1.48^Ca^	35.43±2.31 ^Bb^	53.48±3.21 ^Ba^

Capital letters within columns, lowercase letters within rows with the same letter after the Means±SE did not differ significantly as per one-way ANOVA and Student’s *t*-test (*P* < 0.01), respectively.

Correspondingly for unmated *T*. *japonicum*, similar results were noticed across the host species eggs (24 h exposure: F = 70.41 df = 19, *P*<0.0001; 48 h exposure: F = 347.54, df = 19, *P*<0.0001) as well as the duration exposed (*C*. *cephalonica*: t = -16.65, df = 19, *P*<0.001; *E*. *kuehniella*: t = -13.28, df = 19, *P*<0.001; *S*. *cerealella*: t = -7.8, df = 19, *P*<0.001) (Table 1). There was a considerable reduction in parasitism by unmated *T*. *japonicum* compared to mated at both the exposure duration except *C*. *cephalonica* exposed for 48 h duration.

Differences were observed among the different hosts and exposure duration of the parasitoid in the emergence rates of *T*. *japonicum*. Under mated category, higher percentage *T*. *japonicum* emergence (88.99%) was recorded on *S*. *cerealella* eggs at 24 h exposure, which differed significantly from other hosts (F_2,57_ = 50.25; *P* = 0.0001) and had increased emergence of 30.69% and 23.15% over *C*. *cephalonica* and *E*. *kuehniella*, respectively. Whereas at 48 h, *E*. *kuehniella* recorded the highest emergence of 94.66%, which differed significantly from other host’s eggs (F_2,57_ = 29.37; *P* = 0.0001) and had an increase of 8.66% and 12.27% over *C*. *cephalonica* and *S*. *cerealella*, respectively. The emergence of parasitoids differed significantly with the duration of exposure among all the host eggs used in the experiment. Both *C*. *cephalonica* (t = -1.77; df = 19; *P* = 0.001) and *E*. *kuehniella* (t = -3.164; df = 19; *P* = 0.001) had higher percentage of emergence at 48 h exposure duration, but *S*. *cerealella* (t = -0.988; df = 19; *P* = 0.01) had at 24 h. Whereas, under unmated category, the emergence rates of *T*. *japonicum* also differed significantly among hosts (F_2,57_ = 18.37; *P* = 0.0001). Highest emergence rate was recorded on *E*. *kuehniella* host eggs at both exposure durations (Table 1).

### 3.2 Longevity

There was an increased longevity of *T*. *japonicum* female descendants emerged from different host eggs parasitized by unmated *T*. *japonicum* females when food (honey) was provided at both the duration of exposures *i*.*e*. 24 h (*C*. *cephalonica*: t = -9.895, df = 19, *P* = 0.001; *E*. *kuehniella*: t = -7.909 df = 19; *P* = 0.001; *S*. *cerealella*: t = -10.847; df = 19; *P* = 0.001) and 48 h (*C*. *cephalonica*: t = -9.891, df = 19, *P* = 0.001; *E*. *kuehniella*: t = -7.690 df = 19; *P* = 0.001; *S*. *cerealella*: t = -9.723; df = 19; *P* = 0.001). *Trichogramma japonicum* emerged from *E*. *kuehniella* eggs had significantly highest female longevity when the wasps were provided honey (with food) at 24 h (F_2,57_ = 29.28, *P*<0.0001) and 48 h (F_2,57_ = 34.38, *P*<0.0001) exposure periods. A similar trend was also observed in “without food” at 24 h (F_2,57_ = 21.36, *P*<0.0001) and 48 h (F_2,57_ = 11.60, *P*<0.0001) exposure periods. Similar results were also obtained in mated *T*. *japonicum* female emergence except in the 24 h exposure ‘without food’ treatment where *C*. *cephalonica* and *E*. *kuehniella* were found on par (Table 2).

**Table 2:** The longevity (days) of emerged *Trichogramma japonicum* when host eggs were exposed to mated and unmated female parasitoids for 24 and 48 h under with and without food conditions. Honey was used as a food source for adult parasitoids.

Host	Mated			
	24 h		48 h	
	with food	without food	with food	without food
*Corcyra cephalonica*	6.05±0.18^Ab^	5.50±0.13^Ba^	6.40±0.21^Ab^	5.65±0.20^Bb^
*Ephestia kuehniella*	6.75±0.23^Aa^	5.80±0.15^Ba^	7.35±0.23^Aa^	6.45±0.15^Ba^
*Sitotroga cerealella*	4.85±0.15^Ac^	4.15±0.24^Bb^	5.05±0.15^Ac^	4.50±0.24^Bc^
	**Unmated**			
*Corcyra cephalonica*	7.10±0.16^Ab^	4.85±0.13^Bb^	7.11±0.18^Ab^	4.90±0.14^Bb^
*Ephestia kuehniella*	8.40±0.23^Aa^	5.65±0.15^Ba^	8.47±0.20^Aa^	5.65±0.21^Ba^
*Sitotroga cerealella*	6.25±0.18^Ac^	3.90±0.24^Bc^	6.37±0.17^Ac^	4.15±0.23^Bc^

Capital letters within rows, lowercase letters within columns with the same letter after the Means±SE within each exposure period did not differ significantly as per one-way ANOVA and Student’s *t*-test (*P<* 0.01), respectively.

### 3.3 Parasitism *vs*. days of oviposition by moths

Different host eggs collected from each oviposition day exposed to *T*. *japonicum* for parasitization yielded significant differences. On all days of oviposition, *E*. *kuehniella* eggs has recorded higher percentage of parasitism and differed significantly from other two host eggs (F_5,54_ = 61.26, *P*<0.001; F_5,54_ = 49.93, *P*<0.001; F_5,54_ = 139.56, *P*<0.001; F_5,54_ = 68.87, *P*<0.001; F_5,54_ = 67.70, *P*<0.001; F_5,54_ = 103.69, *P* = 0.001 on 1^st^, 2^nd^, 3^rd^, 4^th^, 5^th^ and 6^th^ day of oviposition, respectively). For *C*. *cephalonica* (χ^2^ = 40.51, df = 5, *P*<0.001), *E*. *kuehniella* (χ^2^ = 43.39, df = 5, *P*<0.001) and *S*. *cerealella* (χ^2^ = 39.57, df = 5, *P*<0.001) eggs, highest parasitisation was recorded on 2^nd^ day of oviposition. Overall, the highest parasitism of *T*. *japonicum* was observed on eggs of *E*. *kuehniella* on its 2^nd^ day of oviposition (79%) (Table 3). Different day of oviposition and the host species were found to be significant individually, but not their interaction by two-way ANOVA (Day of oviposition: F_5,174_ = 87.62, *P* < .0001; Host species: F_2,177_ = 756.76, *P* < .0001; Day of oviposition *Host species: F_10,170_ = 0.39, *P* = 1.06).

**Table 3:** Parasitism (%) of *Trichogramma japonicum* on different factitious host eggs oviposited on different days.

Oviposition period	*Corcyra cephalonica*	*Ephestia kuehniella*	*Sitotroga cerealella*
First day	45.67±1.99d	54.67±1.66d*	31.67±1.34d
Second day	65.00±2.34a	79.00±1.58a*	52.33±1.65a
Third day	57.33±1.30b	73.00±2.08b*	45.00±1.43b
Fourth day	51.00±1.65c	63.33±1.79c*	36.00±1.63c
Fifth day	42.67±1.47d	59.00±1.58cd*	32.33±1.65cd
Sixth day	44.33±1.65d	61.67±1.14c*	33.67±1.16cd

Means±SE in the same column followed by the same letter did not significantly differ according to the Kruskal–Wallis test (p > 0.01).

*indicates a significant difference between values in the same row as per one-way ANOVA (*P* < 0.001).

### 3.4 Flight test

**Fig 1:**
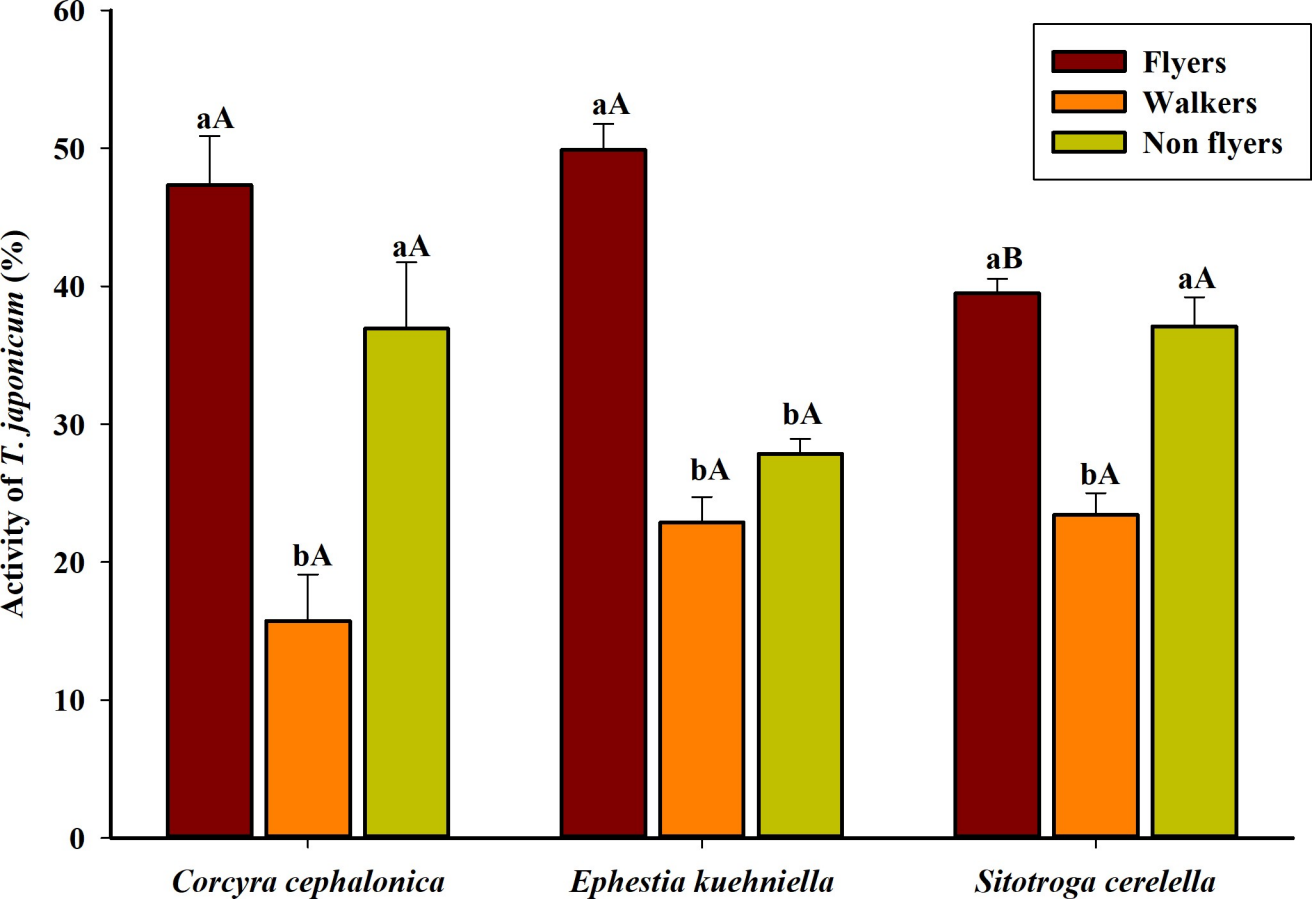
Flight propensity of *Trichogramma japonicum* emerged from the eggs of different factitious hosts. Three flight activities were measured, flyers, walkers and non-flyers. The values are presented as the mean ± standard errors. Treatments were compared using one-way ANOVA (Tukey’s HSD test, *P* < 0.01). The same letter above error bars (capital letters across host species, lowercase letters across flight activities) did not differ significantly (*P* ≤ 0.01).

Among host species used, the proportion of *T*. *japonicum* emerged from only *E*. *kuehniella* eggs had significantly more ‘flyers’ compared to ‘non-flyers’ and ‘walkers’. A higher proportion of flying *T*. *japonicum* was noticed when they were reared on *E*. *kuehniella* eggs (49.90%) and *C*. *cephalonica* eggs (47.33%) compared to *S*. *cerealella* eggs (39.50%) (F_2,18_ = 5.27, *P* = 0.0347). However, non-significant differences were noticed in flight capacity of the parasitoid in terms of ‘walkers’ (F_2,18_ = 2.68, *P* = 0.129) and ‘non-flyers’ (F_2,18_ = 3.42, *P* = 0.08) across different hosts (Fig 1).

### 3.5. Field studies

**Fig 2:**
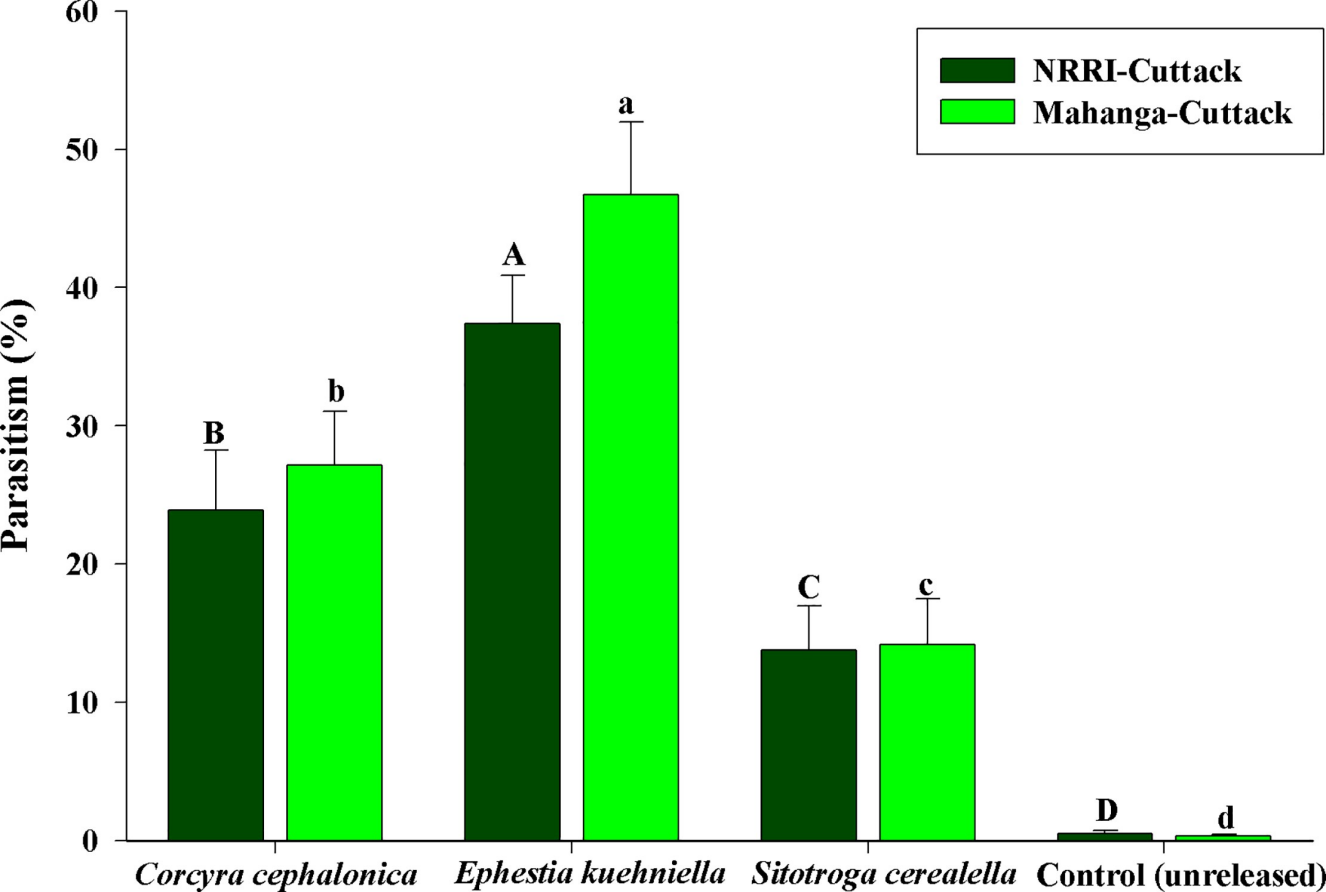
Parasitism of yellow stem borer (YSB) eggs by *Trichogramma japonicum* under field conditions when parasitoid reared on different factitious hosts. Multi-location field studies were conducted at NRRI, Cuttack and Mahanga, Cuttack. The values are presented as the mean ± standard errors. Treatments were compared using one-way ANOVA (Tukey’s HSD test, *P* < 0.01). The same letter above error bars (capital letters for NRRI, Cuttack location, lowercase letters for Mahanga, Cuttack location) did not differ significantly (*P* ≤ 0.01).

A total of 160 egg masses were recollected from both treatment and control plots from both the fields. Data were pooled for further analysis. Results showed significant differences among the treatments in terms of parasitization rates by *T*. *japonicum* at research farm, NRRI, Cuttack (F_3,16_ = 160.50; *P*<0.0001). The higher parasitism (37.41±4.47%) of YSB eggs was observed in the treatment where *T*. *japonicum* reared on *E*. *kuehniella* was released (Fig 2). Similar trend was observed in the results of field experiment conducted at Mahanga, Cuttack, wherein *T*. *japonicum* reared on *E*. *kuehniella* eggs had highest parasitization of YSB eggs (46.71±6.27%) (F_3,16_ = 221.34; *P*<0.0001). Field experiments conducted in the present study suggested that *T*. *japonicum* reared on *E*. *kuehniella* provided more significant field parasitism of a different host (i.e., YSB eggs) in the field.

## 4. Discussion

Host (species, size, age) can impact parasitoids preference and offspring fitness [35]. The laboratory performance of the *Trichogramma* will predict their performance in the field (in suppressing the target pest) [36]. Hence, our study analyzed host dependant changes in the productivity of *T*. *japonicum*, a potential biological control agent released inundatively in India to manage YSB. One of the cornerstone strategies in choosing the most suitable parasitoid for field release is parasitization efficiency. In our study, the parasitism tests revealed a variation in the characteristics of *T*. *japonicum* and all these variations could affect the successful use of *Trichogramma* spp. [36]. Parasitization is one of the crucial characteristics representing the productivity of biological control agents, and it could vary due to differences in the hosts used for rearing. Overall, *E*. *kuhniella* eggs were parasitized the highest compared to others, both at 24 h and 48 h exposure duration.

The mating status would affect the oviposition of parasitoids]. In the current study, we also noticed a lower parasitism rate in the unmated females than mated. Similar results were also obtained by Pratissoli *et al* [37] in a different species, *T*. *acacioi*. Our results showed poor parasitism of *S*. *cerealella* eggs; similar observations were also reported by Parra *et al* [38]. Bai *et al* [39] compared the quality of *T*. *pretiosum* adults reared from different hosts and concluded that it depends on the host’s size from which it emerged. They measured the host egg volume and it was observed to be 0.028±0.003 mm^3^ for *E*. *kuhniella* and 0.022±0.005 mm^3^ for *S*. *cerealella*, but they have not considered *C*. *cephalonica* for the study. However, Consoli *et al* [40] measured the egg size of *C*. *cephalonica* and found it to be approximately 0.036 mm^3^. Apart from egg size, surfaces of host eggs, chorion structures, layers, etc., govern parasitoid development. The chorion surface of *C*. *cephalonica* has a rugged texture but not as evident as in *E*. *kuehniella* [40]; hence, higher parasitization of *T*. *japonicum* on *E*. *kuehniella* eggs was recorded in the present study.

Among different hosts tested, *S*. *cerealella* eggs are the smallest, and there would be depletion of nutrients for embryonic development of parasitoid and subsequently its biology and performance. Host features were previously pointed out as an essential factor for *T*. *remus* parasitism and development by Bueno *et al* [41]. Few studies have already pointed out the differential effects of the age of the host egg [42] on their parasitization, which was also evinced in the current study. Apart from the host egg age, parasitoid age can also influence the rate of parasitization [43].

In the current study, we recorded the highest parasitization on *E*. *kuehniella* eggs. However, the highest parasitoid emergence was recorded on *S*. *cerealella* eggs. The variation in emergence, as well as the varying parasitism rate of *T*. *japonicum*, could be related to the specific morphological characteristics host eggs used, the size and age of the host eggs, and the quality of the eggs for parasitoid development [44]. The emergence of *T*. *japonicum* varied across the host as well as exposure duration. Except for the unmated category in *S*. *cerealella*, emergence decreased when exposure duration was increased. It could be due to continuous and extended contact of parasitoids with host eggs; here, parasitoid may deposit their eggs on to a host that is already parasitized. Further, it results in superparasitism, where some or all of the immature parasitoids are insufficiently nourished and thus fail to develop fully and finally died. This differential emergence could also be due to the different sizes and quality of the hosts. Similar differences in host eggs which, act as an important parameter in *Trichogramma* development and survival, have been reported [40]. Our result about differences in emergence across exposure duration supports the need for inundative release of the parasitoids. Parasitoids will not maintain the sufficient population required to control the pest once emergence has declined in the field.

*Trichogramma japonicum* parasitized the eggs of all host species irrespective of egg’s age, indicating the acceptance of the egg of this species by the parasitoid, however, the level of parasitism varied. Similar findings were also noted by Chen *et al* [45]. In the current study, parasitism increased on the second day of oviposition by host species tested compared to other days. Similar to our results, increased parasitization of 2^nd^ day oviposited *C*. *cephalonica* eggs by *T*. *chilonis* was recorded by Tiwari and Khan [46]. In our study, parasitization abated as the egg-laying (or day of oviposition) advanced. As moth age advances, this could be because they start losing vigor, vitality, and lay fewer eggs. This phenomenon was observed unfavorable for the parasitoid *T*. *chilonis* from the fitness point of view. A parsimonious explanation for the negative relationship between the day of oviposition and parasitoid development could be due to differences in egg nutrients. Parasitoids will not get sufficient nutrients from older eggs. Also eggs may undergo morphological and physiological changes that interfere with their acceptance by the parasitoid [47]. Parasitoids prefer freshly laid eggs to ensure that they get sufficient maximal fecundity [48]. Two hypotheses could explain the interaction between the host egg and parasitoid. Firstly, female parasitoids might have laid eggs similarly irrespective of the host egg laid, but parasitoid larvae could not have developed well in older eggs due to competition with developing native embryos [28,41,49]. Secondly, female parasitoids might sting the host eggs equally regardless of eggs oviposited, but parasitoids would be produced mostly in hosts showing optimal physiology for progeny development [50–52]. Hence, older eggs are considered as a low-quality host food for mass rearing protocols of egg parasitoids [43,49].

Longevity is one of the important quality parameters of a parasitoid. In our study, longevity of adult *T*. *japonicum* has improved with honey, and we anticipate a similar effect in the field when parasitoid feeds on plant nectar. Food for parasitoids is an important factor, and deprived parasitoids will first search for food resources before they search for hosts. In the present study, we have used honey as a food source, and it is expected to have proteins, enzymes, fructose, glucose, and amino acids. Honey deprived *T*. *cacoeciae* only attacked one host patch, whereas fed females were more likely to move to a second host egg patch. Females without supplemental food sources never lived more than 5 d while fed females lived an average of 11 d on honey [53]. Authors further found that longer-lived parasitoids may have greater efficiency in the field and parasitize pest eggs for a more extended period. This feature is essential in biological control programs. In our study, longevity among the descendants was influenced by the mating status of the parasitoid. Pratissoli *et al* [37] also showed the interference of mating on the longevity descendants in *T*. *pretiosum*. Like ours, descendants from unmated parasitoids have recorded 14.27 days’ longevity, whereas 11.33 days for mated parasitoids. Mating is also known to reduce the female lifespan in many insects.

The flight test is a pivotal component to monitor the quality of *Trichogramma* [33,54]. Overall, our study showed that the host insect could affect the flight capacity of *T*. *japonicum* to some extent. Similarly, high flight capacity was observed for *T*. *annulata* and *T*. *atopovirilia* on *C*. *cephalonica*, and *A*. *kuehniella*, respectively [55]. In the present study, *E*. *kuehniella* eggs were much better hosts for *T*. *japonicum* in terms of flight capacity than *S*. *cerealella*. Kolliker-Ott *et al* [56] assessed that the fitness of *Trichogramma* is strongly related to wing shape and size, and they influence dispersal and host location of the parasitoid in the field. Parasitoid wing size is known to be influenced by the host on which they are reared and influences the flight capacity. From our enclosure studies on flight capacity, we cannot exactly mimic the dispersal capacity of the parasitoid in the field condition; however, it has been shown that short-range flight capacity could be an indicator of field dispersal.

In the current study, we have field-tested *T*. *japonicum* reared on different factitious host against its natural host YSB. Achieving higher parasitization of YSB eggs is often tricky due to its very nature of egg mass, consisting of multiple layers of eggs and covered with hairs by the YSB female moth. In our study, *T*. *japonicum* reared from *E*. *kuehniella* eggs had parasitized the highest YSB eggs (37.41±4.47%). On the other hand, meager percent parasitism rates (0.35% ± 0.36%) of YSB eggs were found in the study carried out by Tang *et al* [26]. Further, the authors were also linked poor parasitism to sub-optimum quality of *T*. *japonicum* used. For the first time, the present study reports a comprehensive proof of quality variation based on the hosts used for rearing and their subsequent performance in the field. Mass production systems are crucial for cost efficiency and it depends on the possibility of exploring factitious hosts, which have a low cost of production [57]. We have mass-reared all three host eggs (*C*. *cephalonica*, *E*. *kuehniella* and *S*. *cerealella*) on the natural and locally available diet. In the present study, the total cost production of each factitious host was calculated considering various components of the production cycle. Among the three factitious hosts, cost of production (of one cc) 299 was lowest for *E*. *kuehniella* eggs, i.e. (28.32 INR), followed by *C*. *cephalonica* (29.38 INR) and it was highest *S*. *cerealella* (39.54 INR) [Gowda BG, unpublished observations]. Although the kilogram cost of the natural diet for *S*. *cerealella* (TN-1 paddy grains) was cheaper, producing one cc of eggs requires a higher quantity of diet. Factitious hosts are usually chosen even if they are not the optimal hosts [19]. Hence, to utilize *E*. *kuehniella* as a new host for *T*. *japonicum* its trade-off benefit could be crucial.

One of the key reasons for the failure of biological control programs is the lack of quality parasitoids. The quality-assured parasitoids will have tremendous implications for pest suppression under field conditions than a mere high number of parasitoids. The promotion of biological pest control should be considered on priority due to the effects of pesticides *viz*., residue, resistance, and resurgence. Overall, based on the biological characteristics and field results, the present study comprehensively unveiled that *E*. *kuehniella* is a more suitable host for *T*. *japonicum* in terms of performance both in the laboratory and field. *Cocyra cephalonica* was used *hitherto* for commercial rearing of *T*. *japonicum*. Our study recommends using *E*. *kuehniella* eggs to mass rear *T*. *japonicum* for maximum benefit.

## Supporting information

S1 Fig Flight test unit to measure the flight activity of *T. japonicum*. (a) Inner view of flight test unit (b) Outer view of the flight test unit.
(TIF)
