# Supplementary figures and images for: Performance of Trichogramma japonicum under field conditions as a function of the factitious host species used for mass rearing
Source: PLoS One. 2021 Aug 19;16(8):e0256246. doi: 10.1371/journal.pone.0256246 (PMC8375968; doi:10.1371/journal.pone.0256246)

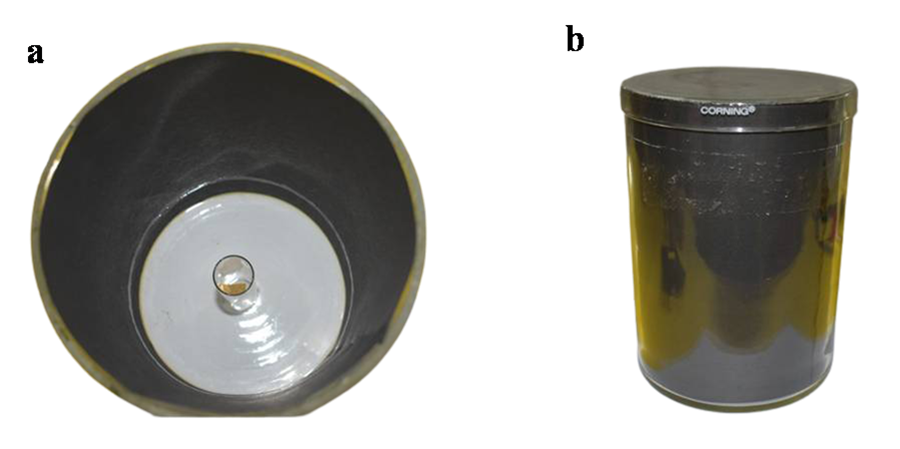

Supplement: S1 Fig — (a) Inner view of flight test unit (b) Outer view of the flight test unit. (TIF) [file pone.0256246.s001.tif]
